# Neural Predictors for the Generalization of Semantic and Phonological Treatment to Discourse Performance in Chronic Post-Stroke Aphasia

**DOI:** 10.1162/NOL.a.27

**Published:** 2025-12-18

**Authors:** Laura Giglio, Leonardo Bonilha, Julius Fridriksson, Sigfus Kristinsson, Roger Newman-Norlund, Chris Rorden, Brielle C. Stark, Janina Wilmskoetter, Dirk B. den Ouden

**Affiliations:** Department of Communication Sciences and Disorders, Arnold School of Public Health, University of South Carolina, Columbia, SC, USA; Department of Neurology, School of Medicine, University of South Carolina, Columbia, SC, USA; Department of Psychology, College of Arts and Sciences, University of South Carolina, Columbia, SC, USA; Department of Speech, Language and Hearing Sciences, Program for Neuroscience, Indiana University Bloomington, Bloomington, IN, USA; Department of Health and Rehabilitation Sciences, Medical University of South Carolina, Charleston, SC, USA

**Keywords:** chronic aphasia, discourse, generalization, hippocampus, lesion-symptom mapping, treatment

## Abstract

Recovery of language function in post-stroke aphasia is affected by many variables, including aphasia severity, age, lesion site and size, and brain health. Semantic and phonological therapies are often used to target naming abilities, and when successful their benefits can extend to discourse production, which has emerged as a promising task to evaluate language processing and recovery in aphasia. Here, after characterizing the lesion and white matter integrity predictors for discourse production before treatment, we asked whether brain integrity at baseline is predictive of treatment generalization to discourse. In a large sample of participants with chronic aphasia (*N* = 88), we ran region-based lesion-symptom mapping on discourse measures (including fluency, sentence processing abilities, and error types) at baseline, on discourse changes following phonological and semantic treatment separately, and at 1 month and 6 months post-treatment. Discourse productivity at baseline was associated with the integrity of regions and white matter tracts in the dorsal stream. Lesions in the hippocampal system and cortical temporal regions were associated with less improvement in discourse following both phonological and semantic treatment. Long-term improvement was instead predicted by the integrity of the fornix and temporal cortical regions, suggesting that while the hippocampal system is important for learning, learned functions rely on connectivity with cortical areas. The results suggest that the generalization of word-level treatment to discourse production is facilitated by an intact hippocampal system in the medial temporal lobe.

## INTRODUCTION

Recovery of language function in post-stroke aphasia is highly variable across individuals and its neural predictors are not yet well understood. The largest recovery gains are usually seen in the first year post-stroke, but, even in chronic aphasia, recovery can continue for years after aphasia onset (Holland et al., 2017; Hope et al., 2013; Johnson et al., 2019; Wilson et al., 2023). Lesion size and site, as well as post-stroke brain health, are contributing factors to recovery outcomes, but much variability is left unexplained (Johnson et al., 2022; Kristinsson et al., 2022; Roth et al., 2023; Thye & Mirman, 2018). Recovery is usually helped by different types of speech-language therapy, among which lexical-retrieval treatment aimed at word production and comprehension is very common (Nickels, 2002; Wisenburn & Mahoney, 2009). While lexical-retrieval treatment usually targets naming performance, it is important to characterize the conditions that allow individuals to extend treatment benefits to other forms of speech, such as discourse, which better reflects functional communication abilities. In this exploratory study, after characterizing the lesion predictors for baseline discourse production in aphasia, we asked whether the generalization of word-level treatment to discourse production is associated with the integrity of specific brain regions.

The current study was part of a large prospective clinical trial. Therapy consisted of 3 weeks of word-level phonological treatment and 3 weeks of semantic treatment in all participants (the order was counterbalanced across participants). Treatment was successful for naming improvements at the group level, both immediately post-treatment and up to 6 months post-treatment (Kristinsson et al., 2023). Milder impairment predicted a good response to semantic treatment, while higher stroke symptom severity was the strongest predictor for the response to phonological treatment (Kristinsson et al., 2021). In the current study, we aimed to expand this perspective by focusing on how treatment generalized to discourse production. Generalization can occur both within-level, such as when lexical-retrieval treatment extends to untreated items, and across-level, when there is a change at a different linguistic level to the focus of treatment. There is limited evidence on across-level generalization, which makes it critical to reach a better understanding of the behavioral and neural mechanisms that allow for generalization (Webster et al., 2015).

### Discourse Production in Aphasia

In the current study, we specifically focused on generalization from treatment targeting lexical-retrieval (i.e., at the level of the word) to discourse. We focused on discourse for several reasons. Most importantly, discourse reflects functional language use better than task assessments, and thus can provide a closer measure of improvement in everyday communication. In addition, by providing a more naturalistic window into language processing, it is less strongly affected by task processing costs and the unavailability of context, which are known to affect lexical retrieval in more constrained naming tasks (Mayer & Murray, 2003). Discourse has the direct benefits of being easy to elicit and requiring less testing time than many assessment tasks, which makes it a good option for clinical practice as well. Challenges with task comprehension and compliance may also affect an accurate estimation of patient abilities. Discourse additionally provides very rich datasets that can be analyzed in a variety of ways to address different questions and, with the development of automated text analysis, do not require unfeasible amounts of manual coding (Giglio et al., 2024; Gleichgerrcht et al., 2021). There are ongoing efforts to standardize clinical discourse practices, which will make it easier to run and analyze discourse tasks in the future (Stark, Bryant, et al., 2023; Stark et al., 2021).

In general, treatment interventions not targeted at discourse performance can extend to improvement in discourse measures (Webster et al., 2015; Whitworth & Webster, 2015). Bird and Franklin (1996) found that participants who improved in picture naming had similar improvements in discourse production. Thompson et al. (2003) found that there were increases in utterance length and in the proportion of grammatical sentences and verbs with correct argument structure in four participants after training on the production of syntactically complex sentences. Verb network strengthening treatment (here used as part of semantic treatment) led to improvement in untrained sentence and discourse contexts, showing a high potential for generalization (Edmonds et al., 2015). Naming ability was seen to correlate with gist production in discourse only in participants with Broca’s aphasia and Wernicke’s aphasia, suggesting that transfer between tasks and generalization may depend on specific individual characteristics (Richardson et al., 2018).

To assess change in discourse production following treatment, we used narrative discourse as elicited through the retelling of the Cinderella story (MacWhinney et al., 2011), which has been found to lead to richer content than picture descriptions and procedural discourse (Stark, 2019). We focused on several discourse variables to assess different aspects of linguistic processes that may have been impacted by stroke and then affected by treatment. The measures we extracted quantify *speech fluency* (such as words per minute [WpM], mean utterance length [MLU]), syntactic and semantic *sentence processing abilities* (e.g., MLU, verbs per utterance [VpU], and propositional density [PD]), and the *types of errors* produced by participants (phonological, semantic or unrelated). We also included the total number of words (Tokens) as a measure of gross output. These measures have been used extensively in previous studies of discourse in aphasia to characterize fluency and aphasia distinctions (e.g., Fromm et al., 2022; Gordon & Clough, 2020; Riccardi et al., 2024). In a study focusing on test–retest reliability of discourse variables, Stark, Alexander, et al. (2023) found that MLU, VpU, WpM, Tokens, and PD were all significantly lower in people with aphasia relative to controls (see also Bryant et al., 2013; Stark, 2019). MLU and WpM were seen to increase from acute to chronic stages of post-stroke aphasia recovery, suggesting that they are suitable measures to investigate discourse performance trajectories in post-stroke aphasia (Brisebois et al., 2022).

All of these discourse variables can in principle be expected to improve after therapy that is focused on lexical production and comprehension, as strengthened lexical access may facilitate or unmask sentence-level syntactic and semantic as well as word-production aspects of discourse. The facilitation in lexical access may surface both in improvements in fluency with more WpM, but also in longer utterances and more complex constructions, due to improvements in verb use (leading to improvements in MLU and VpU). Treatment may additionally improve the accuracy and diversity of words used, both reducing the number of errors and increasing PD. It is important to note that the present analysis on brain integrity predictors for discourse improvement was independent from significant discourse changes at the group-level after therapy. Any post-treatment improvements on these measures may vary widely across participants and therefore be based on individual lesion characteristics.

To summarize, we focused on eight variables extracted from narrative production to index speech fluency and sentence processing abilities in aphasia, as well as error ratios, which were previously found to be affected in aphasia, relative to performance in controls. We aimed to understand which brain structures were important for the generalization of word-level treatment to improvement in these discourse variables, based on individual improvement trajectories.

### Neural Predictors for Aphasia Recovery and Treatment Generalization

Studies in both healthy and post-stroke participants have shown that language processing is supported by a large left fronto-temporal network (e.g., Fridriksson et al., 2018; Giglio et al., 2022; Hu et al., 2023). Impairments in lexical-semantic processing are generally associated with lesions in the ventral stream along the temporal lobe, while impairments in form-to-articulation are associated with dorsal stream lesions spanning the parietal and frontal lobes (Fridriksson et al., 2016, 2018). Phonological and semantic lexical-retrieval treatment is therefore expected to target processes supported by the dorsal and ventral stream, respectively. The neural structures supporting discourse are instead not expected to be limited to a few regions, since discourse relies not only on several linguistic processes working together, such as phonological and semantic processing for lexical retrieval, but also on higher level syntactic and compositional semantic processing, as well as on pragmatic processing. It is therefore expected that discourse engages a large brain network spanning most of the core ‘language’ network. Lesions in different parts of the network, therefore, may affect discourse in different ways. Previous studies found both gray and white matter regions in the dorsal stream to support successful discourse production. In particular, speech fluency measured in WpM was predicted by the integrity of the arcuate fasciculus and gray matter regions active during speech repetitions (Wang et al., 2013). MLU and Tokens were associated with the dorsal stream (Borovsky et al., 2007). The syntactic complexity of discourse produced by individuals with aphasia was related to lesions in the posterior inferior frontal and inferior parietal areas, and by the underlying white matter (Gleichgerrcht et al., 2021). Phonological and semantic errors in discourse instead were seen to be associated with lesions in the dorsal and ventral streams, respectively (Stark et al., 2019).

As for brain structures underlying the response to treatment, lesion site and size are known to be important for severity of the impairment and recovery (Thye & Mirman, 2018; Wilson et al., 2023). Importantly, not just gray matter lesions, but also white matter lesions and structural disconnection are predictive of naming performance and aphasia classification (Bonilha, Rorden, & Fridriksson, 2014; Yourganov et al., 2015). Therefore, in addition to characterizing the relationship between lesion characteristics and discourse performance, we included fractional anisotropy (FA) to capture white matter integrity within spared brain regions. In fact, structural lesions may indirectly affect structural connectivity outside of the lesion, especially in the chronic stage, due to diaschisis or functional disconnection (Bonilha, Nesland, et al., 2014; Gleichgerrcht et al., 2017). Baseline severity and naming skills are predicted by gray matter models of lesion size, but treatment success measured in naming improvement is better predicted by the addition of white matter integrity measured with FA (Meier et al., 2019).

Therefore, we focused on two questions. First, we asked whether gray and white matter integrity are predictive of specific aspects of discourse performance in stroke survivors with aphasia. Based on previous studies, we expected that baseline discourse production would be strongly dependent on dorsal stream regions, with ventral stream regions potentially more relevant for semantic errors and PD. MLU and VpU, reflecting sentence processing abilities, may additionally depend on both ventral and dorsal stream regions, and especially posterior temporal and inferior frontal cortex (den Ouden et al., 2019; Fridriksson et al., 2018; Matchin et al., 2020).

Second, we aimed to understand which baseline lesion characteristics are predictive of outcomes in discourse performance (measured as differences in performance for each discourse variable) after word-level treatment. We focused on changes in discourse measures at 1 and 6 months post-treatment, and on specific improvement following word-level phonological or semantic treatment. Phonological and semantic treatment focus on different aspects of lexical retrieval, and as such they may be more or less successful in participants based on their lesions. The focus on 1 and 6 months post-treatment instead was aimed at understanding whether lesions in certain areas prevent long-term improvement. In particular, we determined whether the same regions underlying baseline performance were associated with lower treatment generalization, or whether separate networks are relevant for treatment generalization. Because of the exploratory nature of this investigation, given the limited evidence on neural predictors for generalization to discourse, we performed whole-brain analyses, rather than focus on specific regions of interest.

In summary, we used several measures of discourse performance to capture different linguistic processes and provide a broader understanding of language function after stroke. We focused on both gray and white matter integrity, using region-based whole-brain lesion-symptom mapping and FA, since they were both previously found to be predictive of impairments in discourse performance and treatment success. Finally, we characterized neural predictors for discourse performance at baseline, as well as for short- and long-term generalization of word-level treatment to discourse performance.

## MATERIALS AND METHODS

### Participants

Participants analyzed in this study were recruited as part of the POLAR (Predicting Outcome of Language Rehabilitation in Aphasia) study (Kristinsson et al., 2021, 2023). Participants were eligible for recruitment if they were between 21 and 80 years old, if they had chronic aphasia (>12 months post-stroke) due to left-hemisphere stroke, were speakers of English as their primary language for over 20 years, were willing and able to provide informed consent and were able to undergo magnetic resonance imaging (MRI) scanning. Participants were excluded if they had severely limited speech output as defined by the Western Aphasia Battery–Revised (WAB-R; Spontaneous Speech score of 0–1; Kertesz, 2007), or severely limited auditory comprehension (WAB-R Auditory Comprehension score of 0–1). The study was carried out at the University of South Carolina and the Medical University of South Carolina, and the study procedures were approved by the Institutional Review Board at both universities. Participants provided informed consent to participate in the study.

For the purposes of this study, we only included participants for whom an MRI scan was available at baseline and discourse samples were available at baseline and post-treatment (see Discourse Procedures and Predictors for more information on discourse features). Therefore, 88 participants with aphasia were analyzed in the study (see Table 1 for baseline demographics and clinical characteristics).

**Table 1.** Baseline demographics and clinical characteristics of the study sample (*N* = 88).

Variable	Range	Mean/Count	*SD*
Age (yr)	29–80	60.6	11.2
Female, No. (%)		34 (38.6)	
Handedness, No. (%)			
Right		77 (86.5)	
Left		11 (11.2)	
Ambidextrous		1 (1.1)	
Education (yr)	12–20	15.6	2.3
Time since stroke onset (mon)	10–241	52	55.2
NIH Stroke Scale score	0–18	6.4	4.0
WAB-R aphasia quotient	14.5–93.1	60	22.5
Baseline PNT correct	0–172	80	61.2
Apraxia of Speech (binary), No. (%)		51 (57.3)	
ASRS Apraxia of Speech severity	0–4	1.63	1.5
Aphasia type by WAB-R, No. (%)			
Anomia		25 (28.4)	
Broca’s		40 (45.4)	
Conduction		12 (13.6)	
Global		4 (4.5)	
Transcortical motor		1 (1.1)	
Wernicke’s		6 (6.8)	
Lesion size (cm^3^)	2.4–458.1	123.2	90.5

*Note*. *SD* = standard deviation; ASRS = Apraxia of Speech Rating Scale; NIH = National Institute of Health; PNT = Philadelphia Naming Test; WAB-R, Western Aphasia Battery–Revised.

### General Study Procedure

Participants first took part in a baseline neuroimaging, medical, cognitive and linguistic assessment. They were then pseudorandomly assigned to one of two treatment groups, to ensure counterbalancing of aphasia severity and baseline naming abilities between the two groups. Both groups followed a 3 week therapy phase that was delivered 5 days per week for an hour per day. After the first therapy phase, there was an outcome assessment, followed by 2 weeks of rest. They then underwent another behavioral assessment preceding the second therapy phase, which was again followed by an outcome assessment. Long-term assessments also took place at 1 and 6 months after therapy. The two therapy phases included phonological and semantic therapy, which the two groups of participants followed in different orders. Phonological therapy included three approaches: phonological components analysis (Leonard et al., 2008), a phonological production task, and a custom-designed computerized phonological judgment task. Semantic therapy consisted of semantic feature analysis (Boyle & Coelho, 1995), a modified version of the Promoting Aphasics’ Communication Effectiveness semantic barrier task (Davis, 2005), and verb network strengthening treatment (Edmonds et al., 2009). (For further discussion of these tasks, see Kristinsson et al., 2021, 2023.)

### Discourse Procedure and Predictors

The discourse samples analyzed here are from the Cinderella narrative. The protocol from AphasiaBank was used. Participants were first reminded of all the major events in the story with a picture book (without words) which they could look at in their own time. They were then asked to retell the events from memory while being recorded for later transcription (instructions are presented in the Supplementary Information, available at https://doi.org/10.1162/NOL.a.27 and in Stark, Bryant, & colleagues’, 2023, best practice guidelines for reporting discourse). Participants repeated the story at all six assessments points, before, during and after treatment phases. If one assessment point was missing for a participant, due to technical problems or degraded audio, that participant was excluded for that time point. This exclusion led to all 88 participants being included for the analysis of discourse at baseline; 83 participants for discourse improvement after phonological treatment; 82 participants for discourse improvement after semantic treatment; 84 participants for discourse improvement 1 month post-treatment; and 78 participants for discourse improvement 6 months post-treatment.

The speech samples at each time point were then transcribed by trained graduate research assistants following Codes for the Human Analysis of Transcripts (CHAT) guidelines and processed using Computerized Language Analysis software (CLAN) for automatic analysis of discourse features (MacWhinney, 2000). The text was divided in utterances (communication units), defined as main clauses with any subordinate clauses. Transcriptions included fillers, repairs, repetitions (that are later excluded by CLAN), as well as errors. Among the discourse features obtained with CLAN, we selected the following measures at baseline for association with lesion characteristics: WpM (excluding repetitions and revisions), VpU (including copulas and excluding modals), PD, MLU (excluding utterances with unintelligible words), and the ratios of phonological errors (per total number of words), semantic errors (per total number of words) and unrelated errors (per total number of words). We excluded ratios between separate measures (e.g., type–token ratio and noun–verb ratio), as both extremes may be related to impairments and as such are not suitable for traditional lesion-symptom mapping analyses. With the exception of error types, which are still coded manually, the calculation of these measures is automated in CLAN, based on transcriptions and utterance divisions following the CHAT protocol (MacWhinney, 2000). PD approximates idea density in speech, and is calculated in CLAN by dividing the number of verbs, adjectives, adverbs, prepositions, and conjunctions by the total number of words (Snowdon et al., 1996; Turner & Greene, 1977). Errors that were real words were categorized as semantic if they were related to the target word (when the target was known); otherwise they were categorized as unrelated errors. Errors were categorized as phonological if they were words and nonwords phonologically related to the target (when the target was known), as well as neologisms and unintelligible words. Therefore, we call phonological errors any sound errors, even without phonological similarity to the target. Behavioral performance at baseline for these features can be seen in Table 2. Intra- and inter-rater reliability was completed using intraclass correlation coefficients on 10% of the discourse samples and was rated good/excellent on all discourse measures.

**Table 2.** Baseline descriptive statistics for the discourse variables of interest.

Variable	Range	Mean	*SD*
No. of words (Tokens)	3–817	177.1	169.4
No. of utterances	2–133	28.2	27.5
Words per minute	2.6–165.6	49.1	34.6
Verbs per utterance	0–2.5	1.0	0.62
Propositional density	0–0.63	0.41	0.13
MLU words	1–13.6	5.9	2.8
Ratio phonological errors (PhonErr)	0–0.73	0.124	0.164
Ratio semantic errors (SemErr)	0–0.20	0.016	0.027
Ratio unrelated errors (UnrelErr)	0–0.20	0.015	0.030

*Note*. Error ratios are calculated out of all words. MLU = mean length of utterance.

To investigate how the baseline integrity of brain structures predicted improvements in discourse after treatment, we focused on the difference in performance after treatment and baseline for all these discourse features. We focused on differences after phonological and semantic treatment, and at 1 month and 6 months post-treatment.

### MRI Acquisition and Preprocessing

MRI data were acquired on a Siemens 3T Prisma scanner with a 20-channel head coil, located at the University of South Carolina or at the Medical University of South Carolina. Participants underwent several MRI sequences, among which T1-weighted, T2-weighted and diffusion-weighted images (DWI) were used for the current study. T1-weighted (MPRAGE) images were acquired with voxel size = 1 mm^3^, field of view (FOV) = 256 × 256 mm, 192 sagittal slices, 9° flip angle, repetition time (TR) = 2,250 ms, inversion time (TI) = 925 ms, echo time (TE) = 4.15 ms, GRAPPA = 2, and 80 references lines. T2-weighted images were acquired with a 3D sampling perfection with application optimized contrasts by using different flip angle evolutions protocol with the following parameters: voxel size = 1 mm^3^, FOV = 256 × 256 mm, 160 sagittal slices, variable flip angle, TR = 3,200 ms, and TE = 212 ms. DWI images were acquired using four-sequence acquisition, with two acquired in the anterior-posterior direction and two in the posterior-anterior direction. The two acquisitions were identical sequences with 1.5 mm isotropic voxels, FOV = 210 × 210 × 120, 90° flip angle, TR = 5,250 ms, TE = 80 ms, 42 diffusion direction encodings, monopolar, slices = 80, averages = 1, diffusion values = 0 s/mm^2^, 1,000 s/mm^2^.

Lesions were manually delineated in native space by a neurologist or by a trained technician blinded to the behavioral data. T2-weighted images were coregistered to the T1-weighted images, to align the lesions to native T1 space. Images were then warped to standard space using enantiomorphic (Nachev et al., 2008) segmentation-normalization (Ashburner & Friston, 2005) from the Clinical Toolbox for SPM (Rorden et al., 2012). The resulting spatial transform was used to reslice the lesion into standard space using linear interpolation, with lesion maps stored at 1 mm isotropic voxel resolution and binarized using a 50% threshold. FA values were obtained from DWI following application of TOPUP and EDDY, using FSL’s DTIFIT (Andersson et al., 2003; Jenkinson et al., 2012; Smith et al., 2006).

### Data Analysis

#### Behavioral analysis

**Figure 1.**
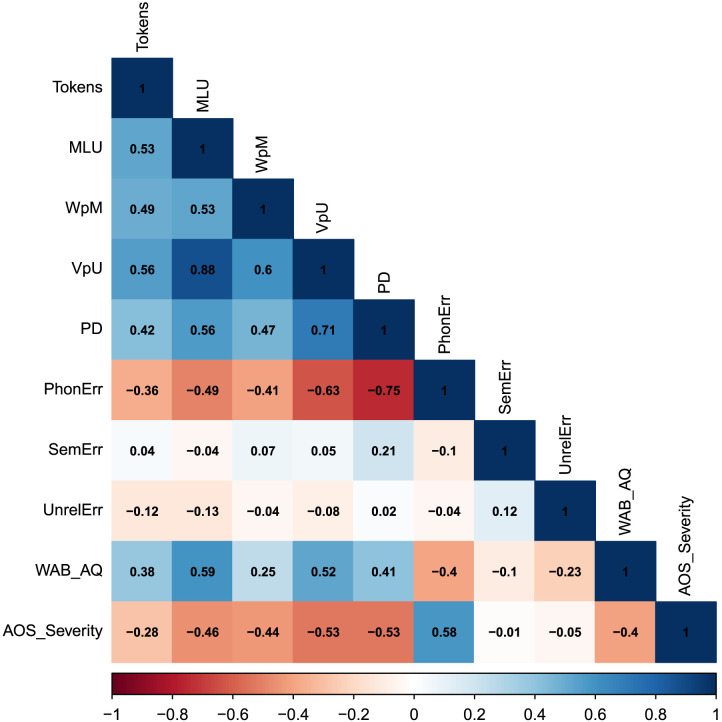
Pearson’s correlations among discourse variables at baseline. Tokens = total number of words; MLU = mean length utterance; WpM = words per minute; VpU = verbs per utterance; PD = propositional density; PhonErr = ratio phonological errors; SemErr = ratio semantic errors; UnrelErr = ratio unrelated errors; WAB-R, Western Aphasia Battery–Revised. AOS_Severity refers to the Apraxia of Speech Rating Scale.

To understand whether discourse performance improved after word-level treatment at the group level, we ran linear mixed-effects models in R on each discourse variable separately (Bates et al., 2015). For each variable, we ran three models with discourse score as the dependent variable, session as a fixed effect, and by-participant random intercepts. In one model, session was coded as 1 month and 6 months post-treatment versus baseline using treatment coding. In another model, we compared performance following phonological treatment to the session just preceding phonological treatment, which was the baseline in a group of participants and a session in-between treatment phases for the other group of participants. In the third model we compared performance following semantic treatment. In these cases, session was also coded with a treatment contrast ([0 1]). For both phonological and semantic treatment, we additionally included treatment order as a fixed effect, coded with a sum contrast ([−1 1]). Note that including age as a covariate to the behavioral models did not affect the effect of treatment on discourse changes, so we did not further control for the effect of age in the brain integrity analyses. Since the different discourse variables analyzed were likely to be correlated with each other, we provide a correlation matrix in Figure 1.

#### Neural predictors for discourse production at baseline

**Figure 2.**
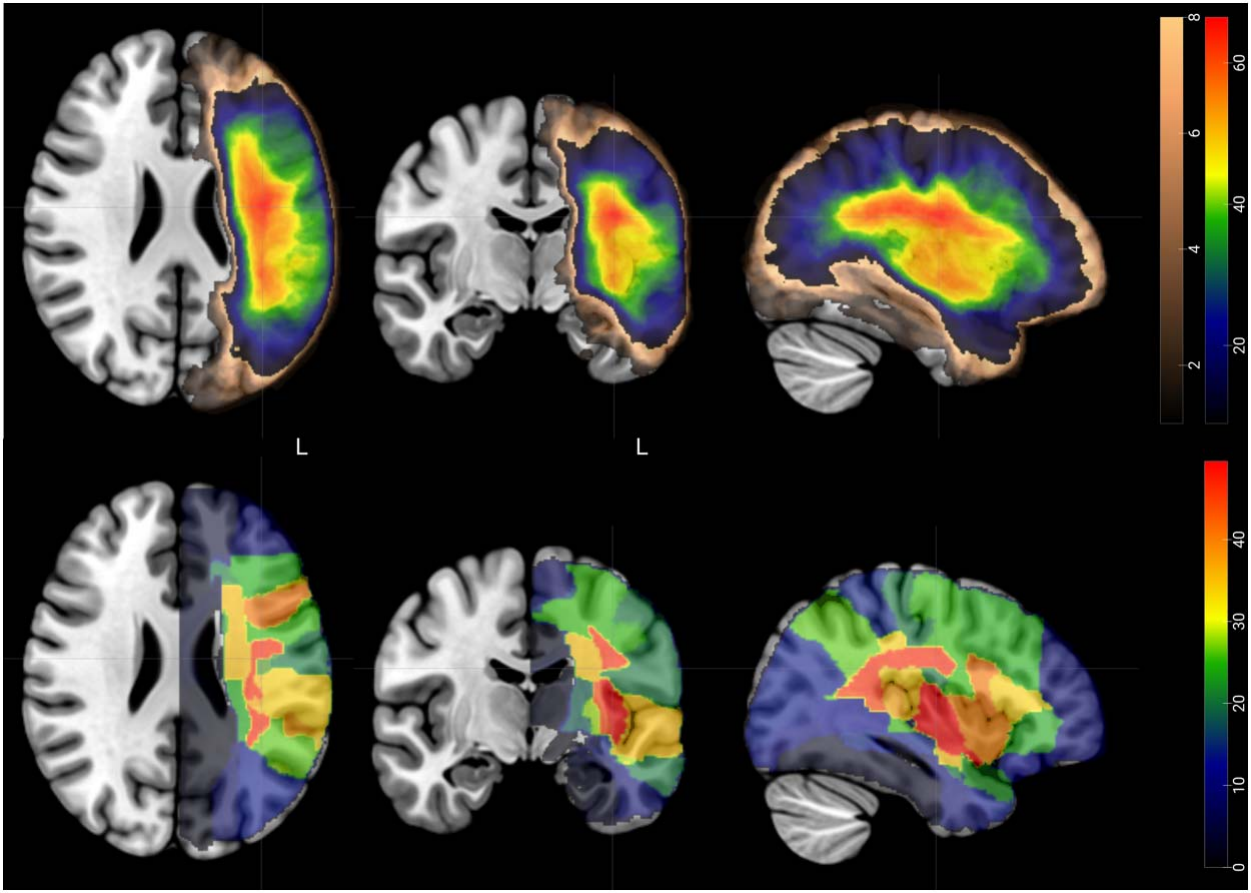
Lesion overlap maps for all participants (*N* = 88) included in the study. Top: Overlap in voxels. The color scales represent the number of participants presenting with a lesion in each voxel. The brown color scale indicates voxels where fewer than nine participants presented a lesion. The cold-to-warm color scale shows voxels most affected by stroke across participants. Bottom: Region overlap map. The color scale indicates the sum of the proportion of regions lesioned in each participant. All colored regions in the bottom row were included in the analysis, as at least 1 voxel per region was lesioned in at least nine participants. Images are shown in radiologic orientation.

To understand whether any localized structural brain damage was predictive of performance on spoken discourse production before treatment, we ran lesion-symptom mapping (LSM) using NiiStat in Matlab2023b (https://github.com/neurolabusc/NiiStat). We ran both region-based LSM (RLSM) using the Johns Hopkins University atlas, which includes both white matter and gray matter regions (Faria et al., 2012). We only selected regions for which at least 10% of the analyzed participants showed a lesion (i.e., 9 for baseline assessments, 8 for discourse improvement). Note that the overlap for regions meant that at least 10% of the participants needed to have a lesion in at least one voxel per region, so more regions survived the overlap threshold than the voxels shown in Figure 2. The RLSM used continuous proportions of how many voxels were lesioned in each region for each participant. A significant relationship between discourse scores and presence of a lesion was determined with permutation testing (5,000 permutations) correcting for multiple comparisons (across regions), while regressing out lesion size. Analyses were one-tailed because injured tissue is predicted to cause poorer performance. The same analysis was also run using FA, as a reflection of white matter integrity, on the Catani atlas which includes a total of 30 tracts (Catani & Thiebaut de Schotten, 2008). The analysis was again one-tailed with decreased FA predicted to cause poorer performance. The FA analysis was run on all participants for whom the DWI data were acquired (83 at baseline, 79 one-month post-treatment, 73 six-months post-treatment, 78 after phonological treatment, 77 after semantic treatment).

#### Neural predictors for discourse outcomes after treatment

To understand how brain integrity at baseline predicted improvement in discourse performance after treatment, we ran the same analysis but now using the difference between discourse measures after treatment and at baseline as behavioral predictors. For general improvement, we focused on differences at 1 month and 6 months after the end of treatment. Therefore, this measure assesses short-term and long-term changes after 6 weeks of sequential phonological and semantic therapy.

Additionally, we asked whether integrity of brain structures predicted specific improvement after phonological versus semantic treatment. For this analysis, we took the difference between discourse performance at the time point just following either type of treatment and the time point just preceding the same treatment. Participants either started with phonological treatment (*n* = 47), or with semantic treatment (*n* = 41). Therefore, type of treatment effects are assessed at different time points for the different groups of participants. To account for these order effects, we regressed out the effect of order from phonological and semantic treatment performance differences using linear regression. We then ran LSM on the residuals. The analysis was then the same as for the baseline predictors.

## RESULTS

### Discourse Performance After Treatment

**Figure 3.**
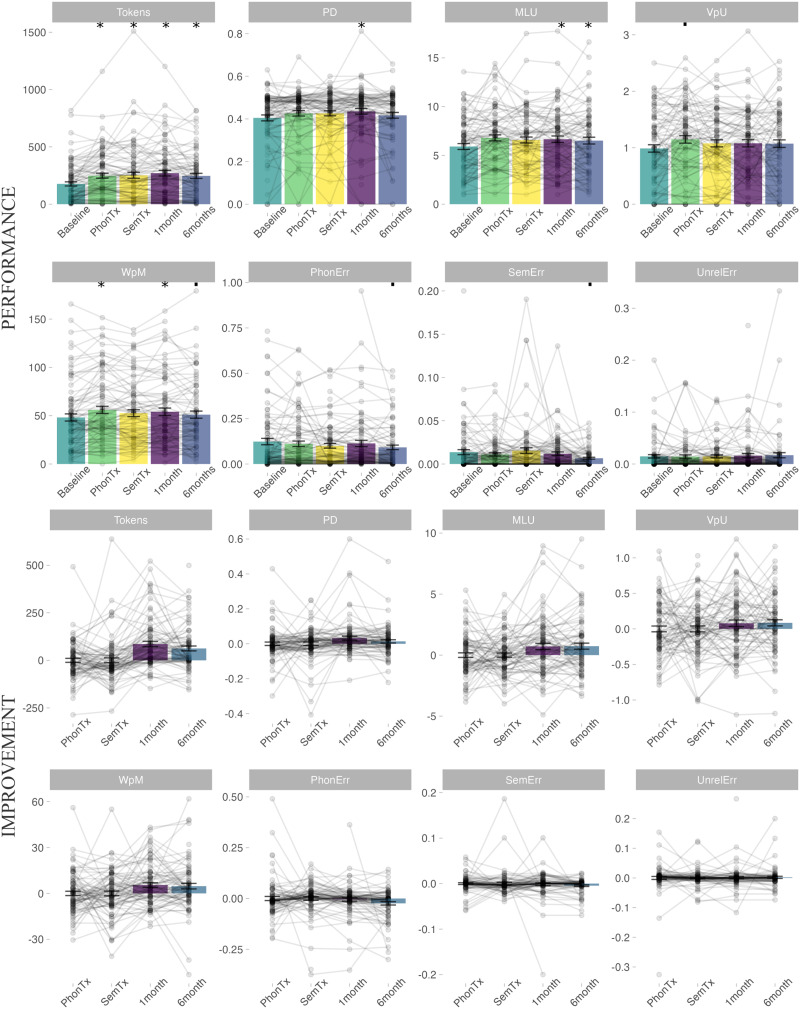
Discourse performance and improvement. Top: Behavioral performance at baseline and after treatment in each of the discourse variables analyzed here. Bottom: Difference between session and baseline (for phonological and semantic treatment, after regressing out treatment order). Note that phonological and semantic treatment were in different orders in two different groups of participants, so the difference is not always against baseline, but against a session in-between treatment phases that is not presented here (see Materials and Methods for more details). Gray dots indicate individual participant scores, linked by gray lines. Not all participants completed all sessions, leading to some unlinked dots. Bars represent the mean and error bars the standard error of the mean. Asterisk (*) indicates *p* < 0.00625 (significant after multiple comparisons correction), ▪ indicates *p* < 0.05 (significant before multiple comparisons correction) in outcome session vs. baseline. PhonTx = phonological treatment; SemTx = semantic treatment.

At baseline, MLU, VpU, PD, Tokens, and WpM were moderately to highly correlated to each other, suggesting they were likely capturing partly similar processes (Figure 1). The number of phonological errors was also correlated with these variables, while semantic and unrelated errors were not correlated with any variables. At the group level, participants improved in most discourse measures after word-level treatment (Figure 3 and Table 3 for statistical information and multiple comparisons corrections). One month after treatment, there was a significant improvement in Tokens, MLU, WpM, and PD. Six months post-treatment Tokens, MLU, WpM, semantic errors, and phonological errors significantly improved. Following phonological treatment, there were significant improvements in Tokens, WpM, and VpU. After semantic treatment, there was a significant improvement in the number of Tokens only. The order of treatment type did not have a significant effect on any of the discourse variables. The behavioral results therefore suggest that there was significant generalization from word-level treatment to discourse performance, although Figure 3 shows large individual variability in patterns of improvement. We thus proceeded to investigate whether lesion characteristics could further explain discourse performance.

**Table 3.** Mixed effects model results for discourse variables that showed a significant improvement following treatment.

Session	Variable	Estimate	*SE*	*t* value	*p* value
1 month	Tokens	88.9	13.8	6.4	0.0001*
	MLU	0.76	0.24	3.1	0.002*
	WpM	5.6	1.66	3.35	0.001*
	PD	0.03	0.01	3.21	0.0016*
	VpU	0.09	0.05	1.83	0.069
6 months	Tokens	63.2	14.2	4.46	0.001*
	MLU	0.72	0.25	2.86	0.005*
	WpM	4.6	1.7	2.7	0.007
	VpU	0.087	0.05	1.81	0.07
	SemErr	−0.006	0.0027	−2.36	0.02
	PhonErr	−0.025	0.009	−2.7	0.008
PhonTx	Tokens	33.1	11.0	3.01	0.003*
	WpM	4.09	1.47	2.79	0.006*
	VpU	0.1	0.04	2.46	0.016
	PD	0.018	0.01	1.78	0.078
	MLU	0.35	0.18	1.83	0.067
SemTx	Tokens	36.9	13.1	2.8	0.0062*

*Note*. Phonological and semantic treatment were in different orders in two different groups of participants, so the difference is not always against baseline, but against a session in between treatment phases (see Materials and Methods for more details). After multiple comparisons correction, only *p* values < 0.00625 are significant (marked with *). Tokens = total number of words; MLU = mean length utterance; WpM = words per minute; PD = propositional density; VpU = verbs per utterance; SemErr = ratio semantic errors; PhonErr = ratio phonological errors; PhonTx = phonological treatment; SemTx = semantic treatment.

### Neural Predictors of Discourse Performance at Baseline

**Figure 4.**
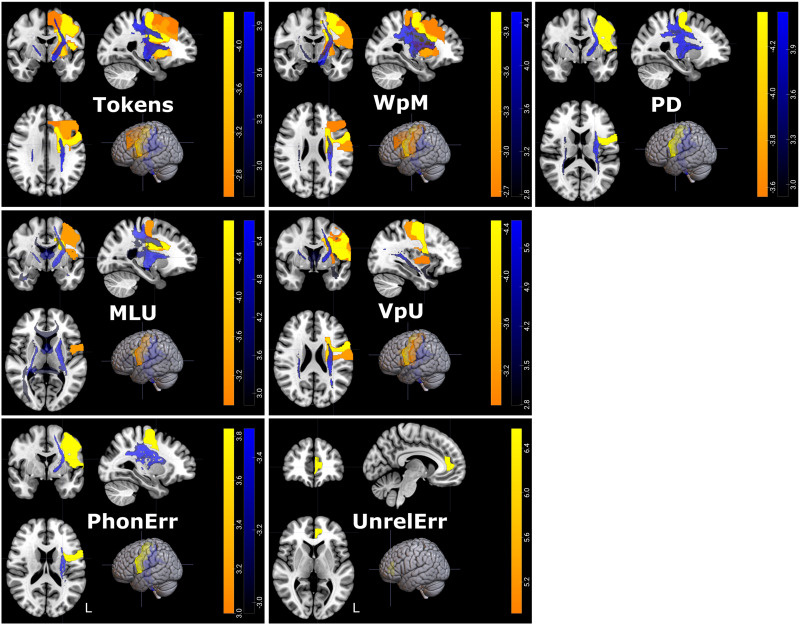
Region-based lesion-symptom mapping (RSLM) and fractional anisotropy (FA) results for discourse performance at baseline. Significant regions are presented in warm colors for RLSM and in blue for FA. Colorbars indicate *z* scores per region. Regions were significant with one-tailed *p* < 0.05, corrected for multiple comparisons. Images are shown in radiologic orientation.

Figure 2 shows the lesion overlap map for the stroke survivors in this study. Regions of highest overlap were in the white matter between the frontal, superior temporal, and parietal lobes. We found several associations between lesion site and white matter integrity and discourse production at baseline (Figure 4 and Table 4).

**Table 4.** Region-based lesion symptom mapping (RLSM) and franctional anisotrpy (FA) mapping of discourse features at baseline.

Discourse feature	Neural predictor	ROI/WM tract	*Z* score
Tokens	RLSM	superior frontal gyrus left (posterior)	−2.61
		middle frontal gyrus (posterior) left	−3.03
		precentral gyrus left	−4.21
		putamen left	−3.85
		globus pallidus left	−3.33
		superior corona radiata left	−4.31
		posterior limb of internal capsule left	−3.19
		external capsule left	−3.16
		ansa lenticularis left	−3.06
	FA	corticospinal left	3.99
		internal capsule	3.58
MLU	RLSM	precentral gyrus left	−3.29
		superior corona radiata left	−4.78
	FA	anterior segment left (arcuate)	3.18
		corpus callosum	3.26
		corticospinal left	5.73
		fornix	4.39
		internal capsule	5.17
		long segment left (arcuate)	4.42
		inferior longitudinal fasciculus right	3.08
		optic radiations right	3.36
		superior cerebellar pedunculus right	3.52
WpM	RLSM	middle frontal gyrus (posterior segment) left	−2.71
		postcentral gyrus left	−2.92
		precentral gyrus left	−4.05
		putamen left	−3.05
		superior corona radiata left	−3.97
		posterior limb of internal capsule left	−2.96
		external capsule left	−2.79
	FA	anterior segment left (arcuate)	3.08
		corticospinal left	4.50
		internal capsule	3.15
		long segment left (arcuate)	4.59
VpU	RLSM	postcentral gyrus left	−3.25
		precentral gyrus left	−4.47
		superior corona radiata left	−4.01
		external capsule left	−2.93
	FA	anterior segment left (arcuate)	3.77
		corticospinal left	6.09
		fornix	3.46
		internal capsule	4.96
		long segment left (arcuate)	5.24
		uncinate left	3.02
		optic radiations right	2.95
		superior cerebellar pedunculus right	2.98
PD	RLSM	precentral gyrus left	−4.35
	FA	corticospinal left	4.15
		internal capsule	3.46
		long segment left (arcuate)	2.99
PhonErr	RLSM	precentral gyrus left	3.83
	FA	corticospinal left	−3.48
		long segment left (arcuate)	−3.17
UnrelErr	RLSM	rostral anterior cingulate gyrus left	6.57

*Note*. Negative *Z* scores for the lesion modality reflect a negative relationship between presence of a lesion and behavioral performance (presence of a lesion corresponds to increased signal, so we expected increased signal to correspond to worse behavioral performance). A positive relationship between FA and behavioral scores is instead reflected in positive *Z* scores (since worse performance is expected with decreased FA). Opposite *Z* scores are considered for error ratios, since higher error ratio corresponds to worse behavioral performance. ROI = regions of interest; WM = white matter.

The total number of Tokens produced was associated with baseline lesions in the left superior frontal gyrus, middle frontal gyrus, precentral gyrus, putamen, globus pallidus, superior corona radiata, posterior internal capsule, and external capsule. Fewer Tokens were also associated with decreased FA in the left cortico-spinal tract and the internal capsule.

RLSM indicated that lower MLU was associated with lesions in the left precentral gyrus and in the superior corona radiata. Lower MLU was also associated with decreased FA in the left anterior segment of the arcuate fasciculus, left corticospinal tract, corpus callosum, fornix, internal capsule, left long segment of the arcuate, right inferior longitudinal fasciculus, right optic radiations, and right superior cerebellar peduncle.

Fewer WpM were associated with lesions in the following regions: left precentral and postcentral gyri, left posterior middle frontal gyrus, left superior corona radiata, left posterior internal capsule, left external capsule, and left putamen. Fewer WpM were also associated with lower FA in the left anterior segment of the arcuate fasciculus, left corticospinal tract, internal capsule, and left long segment of the arcuate.

Fewer VpU were associated with the left precentral and postcentral gyrus, left superior corona radiata, and the left external capsule in the region-based analysis. Fewer VpU were associated with lower FA in the left anterior and long segments of the arcuate, left corticospinal tract, fornix, internal capsule, left uncinate, right optic radiations, and right superior cerebellar peduncle.

Lower PD was also associated with lesions in the left precentral gyrus and with lower FA in the left corticospinal tract, internal capsule and left long segment of the arcuate fasciculus.

The ratio of phonological errors was associated with lesions in the left precentral gyrus with RLSM and with lower FA in left corticospinal tract and in the left long segment of the arcuate fasciculus. The ratio of unrelated errors was associated with lesions in the left rostral anterior cingulate gyrus with RLSM, but no significant tracts with FA. The ratio of semantic errors was instead not significantly associated with lesions in any specific regions.

Therefore, the RLSM and FA analyses converged in most cases in showing that most discourse measures at baseline were affected by the integrity of the precentral gyrus and neighboring regions, as well as of the underlying white matter tracts. The FA results additionally often identified right-hemisphere tracts to be related to discourse performance.

### Neural Predictors of Long-Term Discourse Outcomes After Naming Intervention

**Figure 5.**
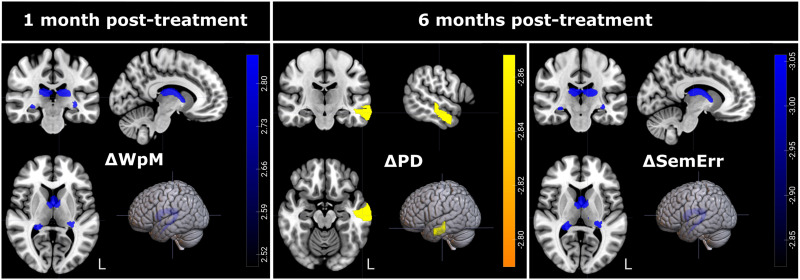
Neural results for discourse improvement 1 month and 6 months after treatment. Figure show regions significantly associated with change in discourse measures as indicated by the RLSM analysis in yellow, and by the FA analysis in blue. Regions were significant with one-tailed *p* < 0.05, corrected for multiple comparisons. Colorbars indicated *z* scores. Images are shown in radiologic orientation.

LSM on discourse improvement also revealed a few regions that predicted less improvement after treatment if lesioned or with lower FA at baseline (Figure 5 and Table 5). One-month post-treatment, less improvement in WpM was predicted by lower baseline FA in the fornix. No regions were associated with improvement in Tokens, MLU, VpU, PD, or error types 1 month post-treatment.

**Table 5.** Results for the region-based lesion symptom mapping and fractional anisotropy mapping to improvement in discourse features after treatment.

Discourse feature	Time point	Neural predictor	ROI/WM tract	*Z* score
MLU	PhonTx	FA	inferior longitudinal fasciculus left	2.93
			optic radiation left	2.86
WpM	1 month post	FA	fornix	2.86
VpU	PhonTx	RLSM	middle temporal gyrus left	−3.38
			pole of middle temporal gyrus left	−3.06
			inferior temporal gyrus left	−3.21
			hippocampus left	−3.04
			posterior middle temporal gyrus left	−3.03
		FA	inferior longitudinal fasciculus left	3.33
			optic radiation left	3.11
			posterior segment left (arcuate)	3.12
PD	6 month post	RLSM	middle temporal gyrus left	−2.88
	SemTx	RLSM	inferior temporal gyrus left	−3.67
			parahippocampal gyrus left	−4.67
			entorhinal area left	−4.27
			amygdala left	−4.93
			thalamus left	−3.52
			fornix (cres) / stria terminalis left	−3.46
			optic tract left	−3.65
PhonErr	PhonTx	RLSM	parahippocampal gyrus left	5.76
			amygdala left	4.72
SemErr	6 month post	FA	fornix	−3.06
UnrelErr	PhonTx	RLSM	posterior cingulate gyrus left	3.12

*Note*. Negative *z* scores for the lesion modality reflect a negative relationship between presence of a lesion and behavioral performance. (Presence of a lesion corresponds to increased signal, so we expected increased signal to correspond to worse behavioral performance.) A positive relationship between FA and behavioral scores is instead reflected in positive *z* scores, since worse performance is expected with decreased FA. Opposite *z* scores are considered for error ratios, since higher error ratio corresponds to worse behavioral performance.

At 6 months post-treatment, less improvement in semantic errors was predicted by reduced FA in the fornix. Less improvement in PD was predicted by lesions to the left middle temporal gyrus with RLSM. No further associations were found between regions and improvement in Tokens, MLU, VpU, or phonological or unrelated errors at 6 months post-treatment.

### Neural Predictors of Short-Term Discourse Outcomes After Semantic and Phonological Treatment

**Figure 6.**
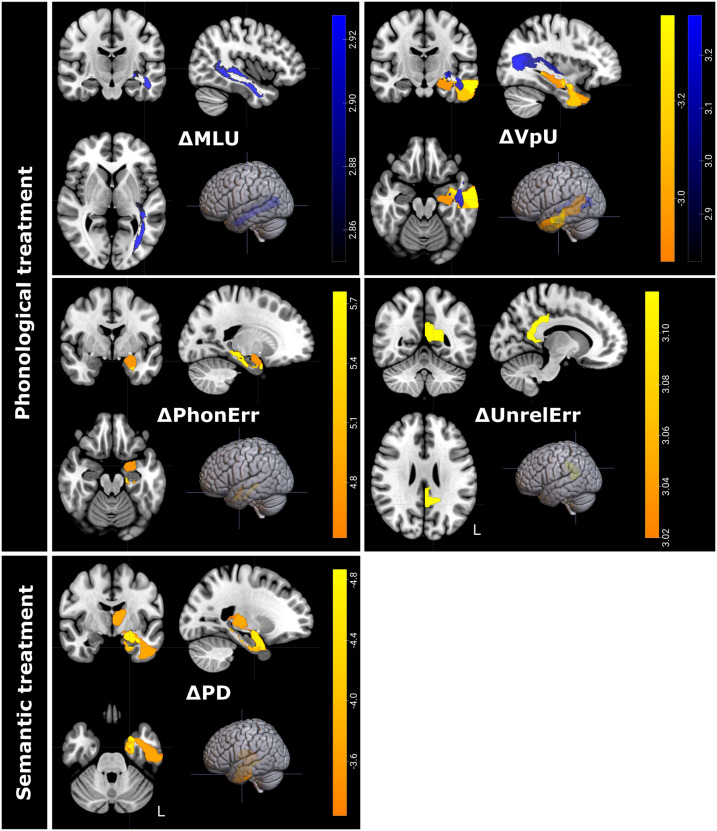
Neural results for discourse improvement after semantic and phonological treatment. RLSM results are presented in warm colors, while FA results are presented in blue colors. Significant voxels are shown in different colors. Regions and voxels shown were thresholded with one-tailed *p* < 0.05, corrected for multiple comparisons. Colorbars indicate *z* scores. Images are shown in radiologic orientation.

Improvement following phonological and semantic treatment was predicted most consistently by the integrity of temporal regions (Figure 6 and Table 5). Smaller improvement in PD following semantic treatment was predicted by damage to left medial temporal lobe structures, and in particular to the inferior temporal lobe, parahippocampal lobe, entorhinal cortex, amygdala, thalamus, fornix, and optic tract with RLSM. No regions significantly predicted improvement following semantic treatment in Tokens, MLU, WpM, VpU, or error types.

Following phonological treatment, a smaller increase in VpU was predicted by lesions in the left middle and inferior temporal lobe extending anteriorly, and by damage to the left hippocampus. Lower baseline FA in the left inferior longitudinal fasciculus, optic radiation, and posterior segment of the arcuate fasciculus also predicted less improvement in VpU. Smaller MLU improvement was predicted by lower FA in the left inferior longitudinal fasciculus and in the optic radiation. Less improvement in the ratio of phonological errors was related to lesions in the left parahippocampal gyrus and amygdala with RLSM. Less improvement in the ratio of unrelated errors was predicted by damage to the left posterior cingulate gyrus with RLSM. No regions predicted improvement in Tokens, WpM, PD, or semantic errors following phonological treatment.

Therefore, the integrity of brain structures at baseline was predictive only of PD improvement after semantic treatment, and of improved MLU, VpU, and phonological and unrelated error ratios following phonological treatment. For both types of treatment, the integrity of the left temporal lobe and especially medial temporal structures was related to better outcomes.

## DISCUSSION

In this study, we aimed to uncover brain lesion characteristics that are associated with impaired discourse production at baseline in left-hemisphere stroke survivors with aphasia, and to find baseline neural predictors for discourse outcomes following naming treatment. We focused on productive discourse measures that reflect fluency (Tokens, WpM, MLU), syntactic processing (MLU, VpU), and content richness (PD), as well as on different types of error ratios (phonological, semantic, and unrelated). At the group-level, performance improved in fluency and complexity of the discourse following phonological treatment and at longer intervals after treatment. The neural results indicated that, at baseline, injury in the precentral gyrus and underlying dorsal white matter was predictive of worse performance for most discourse features. The response to phonological and semantic treatment, as measured in changes to the same discourse variables, was most consistently associated with the brain integrity of the temporal lobe and in particular medial temporal structure. Neural predictors for long-term outcomes in discourse performance were associated with the integrity of the fornix and the temporal lobe.

### Neural Predictors for Baseline Discourse Performance

At baseline, measures of fluency and sentence processing, such as MLU, WpM, and VpU, as well as PD, were related to the integrity of dorsal stream regions. In particular, lesions in the precentral and postcentral gyri and in the superior longitudinal fasciculus were most consistently associated with impaired language productivity. These regions are considered to be part of the dorsal stream, which is associated with performance on phonological processing (Hickok & Poeppel, 2007) and has been argued to support form-to-articulation for word production (Fridriksson et al., 2016). Speech fluency and several measures of sentence and discourse production have been linked to the integrity of dorsal gray matter and white matter regions before (Alyahya et al., 2020; Ding et al., 2023; Fridriksson et al., 2018; Gleichgerrcht et al., 2021; Mirman et al., 2019). Therefore, the ability to produce longer sentences, the ease of word production, and the ability to encode verbs and propositions, are all dependent on regions associated with the dorsal stream.

A previous study found that lesions in the dorsal stream were related to motor speech impairments, but higher level linguistic impairments, such as for grammatical processing or naming, were seen to rely on both dorsal and ventral stream regions (Fridriksson et al., 2018). This suggests that the impairments seen in discourse production here may be strongly affected by difficulties with motor encoding and articulation, which could also in part drive the collinearity between these discourse measures. The importance of other areas for syntactic and semantic processes as captured by VpU and PD is here only visible through white matter integrity of, for example, the arcuate fasciculus and the uncinate fasciculus. Degeneration of these white matter tracts suggests reduced efficiency in connectivity among left and right cortical regions, which may affect the successful encoding of discourse at several linguistic levels, as evident from the link with most measures of discourse (Bonilha, Rorden, & Fridriksson, 2014; Newman-Norlund et al., 2024; Wilmskoetter et al., 2022). The amount of disconnection between regions, therefore, seems to be key for discourse performance. It should be noted that while the FA results especially may have been influenced by other factors, such as age, it was not our goal to isolate neural predictors that are strictly lesion related, but rather to understand the importance of post-stroke structural integrity for discourse production and treatment generalization, regardless of the specific source for decreased integrity.

Different error types were related to different neural sources. There were fewer phonological errors with intact dorsal stream regions analogous to the other discourse measures discussed above. Unrelated errors were instead associated with the integrity of the anterior cingulate cortex. The functional source of real-word errors that are neither semantically nor phonologically related to the target word is inherently unclear and likely varied, and therefore unlikely to have a single neural substrate. Previous studies that focused on unrelated errors in both discourse and naming tasks found associations of unrelated errors with cortical temporal lobe integrity, which was interpreted as unrelated errors deriving from semantic deficits (Meier et al., 2022; Stark et al., 2019). The current results suggest instead a self-monitoring origin for these types of errors, where individuals with damage to the anterior cingulate cortex may be less able to identify and correct their speech errors (Gauvin et al., 2016; Mandal et al., 2020; McCall et al., 2022; Nozari et al., 2011; Teghipco et al., 2023).

### Neural Predictors for the Generalization of Phonological and Semantic Treatment to Discourse Production

The generalization of phonological and semantic treatment to discourse production was predicted mostly by regions in the temporal lobe. In particular, following phonological treatment increases in VpU were predicted by the integrity of the hippocampus and the inferior temporal lobe, increases in MLU were predicted by the integrity of the inferior longitudinal fasciculus, and a decrease in phonological error ratio was predicted by the integrity of medial temporal structures and the temporal pole. Increased PD following semantic treatment was predicted by the integrity of the entorhinal cortex and the parahippocampal gyrus. Therefore, differences between pre- and post-treatment discourse production were dependent on the integrity of the medial temporal lobe for both types of treatment. Lesions in these areas led instead to worse outcomes for different types of discourse variables following each treatment type. Phonological treatment was associated with benefits in sentence processing, as well as phonological and unrelated error ratios. Semantic treatment was instead associated with positive outcomes in propositional density in relation with specific lesion characteristics.

Treatment, therefore, seems to generalize to improved discourse performance in speakers with intact medial and inferior temporal areas. The hippocampal system in the medial temporal lobe is critical for rapid learning of new information, which is gradually integrated with existing knowledge by driving long-term memory consolidation in neocortical systems (Eichenbaum, 2017; McClelland et al., 1995, 2020). The hippocampus has been implicated in several processes, from declarative memory consolidation and motor sequence learning (Jacobacci et al., 2020; Takashima et al., 2006, 2009) to sentence processing (Covington & Duff, 2016; Frankland & Greene, 2020; Piai et al., 2016). The entorhinal cortex is located adjacent to the hippocampus and it provides the majority of cortical input to the hippocampus, while receiving input from many subcortical and cortical structures (Garcia & Buffalo, 2020). The entorhinal cortex supports relating new information with existing knowledge. The parahippocampal gyrus receives input from the hippocampus and connects with temporal and frontal cortical structures (Aggleton, 2012). It is therefore not surprising that in order for the benefits of naming intervention to generalize to connected language production this episodic memory system must be intact. Interestingly, these results imply that the right-hemisphere medial temporal structures are not fully able to take over the function from the damaged left structures, possibly due to the importance of the existing connectivity of the left hippocampal system with left-lateralized language areas. Damage to the fornix and the inferior longitudinal fasciculus, both connecting to the hippocampal system (Aggleton, 2012; Latini et al., 2017), was in fact also associated with less improvement in discourse following therapy in the present study.

The integrity of the hippocampus and its connectivity have been found to be relevant for post-stroke memory and language functions, as well as recovery, in previous studies as well. Following stroke, memory ability and improvements are associated with connectivity of the hippocampus with several cortical regions, such as inferior parietal lobule, parahippocampal cortex and prefrontal cortex (Jung et al., 2021; Lu et al., 2024; Yang et al., 2014). Gray matter volume in the hippocampus and fornix at 3 months post-stroke was associated with improvements in long-term and short-term memory at 12 months post-stroke (O’Sullivan et al., 2022). The integrity of hippocampal structures was also seen to be related to treatment gains in post-stroke aphasia recovery (Goldenberg & Spatt, 1994). Hippocampal gray matter volume in the acute stage predicts language improvement from the acute to the chronic phase and is correlated with statistical learning abilities in people with aphasia (Schevenels et al., 2022). Naming outcomes after phonological naming treatment in chronic aphasia are also related to the integrity of the hippocampus (Meinzer et al., 2010), and functional activation of the hippocampus is seen to be related to short-term naming treatment success (Menke et al., 2009). A recent study on the semantic variant of primary progressive aphasia critically found that the integrity of hippocampus was associated with post-treatment naming accuracy of both treated and untreated items, suggesting that the hippocampus is important for the generalization of learning to untreated items (Dial et al., 2023). Dial et al. (2023) suggested that the hippocampus may support the consolidation of strategies learned during the intervention, which can also facilitate word retrieval for untrained items.

In the current study, the integrity of the hippocampal system resulted in improved propositional density following semantic treatment. Propositional density reflects content richness and is likely to increase with higher accuracy and precision in naming. Among the damaged regions negatively associated with the response to semantic treatment was the entorhinal cortex, together with the parahippocampal gyrus and the fornix. The right entorhinal cortex was previously reported to show increased functional neural activation during naming following semantic treatment in a patient with fluent aphasia (Fridriksson et al., 2007). Cortical thickness in the entorhinal cortex and parahippocampal cortex was also correlated with the semantic precision of words produced in picture descriptions in patients with the semantic variant of primary progressive aphasia (Quaranta et al., 2022). Semantic treatment may rely on the ability to strengthen the relationship with existing semantic knowledge and relations. For this to work, existing semantic knowledge, encoded in several brain regions and accessible via the anterior temporal lobe, needs to be spared, together with the medial temporal lobe system that allows for the integration of incoming information in this existing network (Lambon Ralph et al., 2017; McClelland et al., 2020). Therefore, it seems that for semantic treatment to generalize to discourse production, the ability to integrate incoming information into existing knowledge structures in language regions in the neocortex needs to be available.

The hippocampal system was also critical in the generalization of phonological treatment to VpU and phonological errors. It is interesting that, although verbs were only a small percentage of the treated items (Kristinsson et al., 2023), damage to the hippocampus, middle temporal lobe and posterior temporal lobe were associated with less improvement in VpU. VpU do not just reflect ease with verb production, but they can also reflect syntactic complexity, since more verbs per utterance imply a more complex sentence structure, including, for example, embeddings. Verb encoding has been associated with posterior temporal and inferior parietal structures (den Ouden et al., 2009, 2019; Elli et al., 2019; Meltzer-Asscher et al., 2013, 2015). Damage to both the hippocampus and posterior temporal structures therefore may have prevented the long-term consolidation of functions targeted by treatment in cortical temporal areas as facilitated by the hippocampus. More successful access to word-form representations and increased phonological awareness may have had positive effects both for sentence processing and verb encoding and for phonological error ratios. The importance of the inferior longitudinal fasciculus for MLU following phonological treatment may fall within the same context, where the connectivity of the hippocampal system with language-relevant cortical areas is key for the generalization and consolidation of the intervention. The structural connectivity of the temporal lobe was previously found to be related to improvement in naming following semantic and phonemic anomia treatment (Bonilha et al., 2016).

### Neural Predictors for the Long-Term Generalization of Word-Level Treatment to Discourse Production

At 1 month and 6 months post-treatment, long-term changes in discourse production were associated with the integrity of the fornix and the temporal lobe. The integrity of the middle temporal gyrus was related to positive changes to PD 6 months post-treatment, while positive changes to WpM 1 month post-treatment and to semantic errors 6 months post-treatment were predicted by baseline FA in the fornix, which carries hippocampal projections to thalamus and prefrontal cortex (Aggleton, 2012). Therefore, the hippocampal formation was seen to be related to treatment outcomes only right after treatment, as assessed after phonological and semantic treatment, but not in long-term outcomes. The hippocampus is thus critical for learning, but its involvement may be less linearly related to long-term outcomes, which may rely instead on learned procedures through connectivity with cortical regions. In a previous study, activity in the hippocampus was found to be associated with worse naming and longer reaction times after treatment in the presence of small left inferior frontal gyrus lesions, suggesting that for treatment to be successful consolidation in relevant areas and reduced reliance on the hippocampus needs to occur (Gore et al., 2022).

### Limitations

The finding of similar regions being associated with performance in most discourse variables at baseline suggests that the different discourse measures may be essentially capturing the same underlying impairment. For example, rather than capturing differences in syntactic processing or the ability to encode informative sentences, variance in VpU and PD may be strongly affected by the ability of participants to articulate words. Effectively, WpM, MLU, VpU, and PD at baseline may all highlight regions associated with speech fluency, as also suggested by the correlations between these measures. A previous discourse study also found similar lesion predictors for both MLU and numbers of words spoken (Borovsky et al., 2007). Difficulty with articulating words may thus mask variability in sentence processing in discourse production. Following treatment, these variables instead result in some differences in the relationship with brain integrity, possibly because the differences between two time points removes baseline fluency effects and may thus capture more variable-specific variance. Even though baseline discourse measures may be less broadly informative than anticipated in samples with many nonfluent participants, the finding of consistent regions associated with improvement in discourse following naming treatment highlights that discourse analysis provides a powerful tool to tap into the relationship between lesions, naming treatment, and generalization for communication. Critically, discourse measures are sensitive enough to show different effects for phonological and semantic treatment within a single relatively short task.

In this study, we assessed narrative discourse performance using the Cinderella story. The Cinderella elicitation method has been broadly used in aphasia research, and was recently rated as “valid for collecting and generating spoken discourse samples” by 70% of responders to a global survey of aphasiology researchers (Stark, Bryant, et al., 2023, p. 772). By being long relative to other discourse elicitation methods, the Cinderella story constitutes a powerful elicitation task to obtain a discourse sample that spans several linguistic abilities for participants to be able to retell most events. Narrative storytelling also provides more and richer data than picture descriptions or procedural discourse (Alyahya et al., 2020; Stark, 2019). However, performance may be confounded by the familiarity of the speaker to the story and by their memory resources. Performance over time may also be affected by having to retell the story multiple times (in treatment studies that monitor progress over time, such as here), by motivation loss, or by learning what aspects of the story to focus on. Therefore, analysis of a wider range of discourse tasks (descriptive, procedural, conversational, etc.) may help to improve our understanding of the neural structures that support discourse production as well as treatment-induced changes to discourse production, and of the relevance of hippocampal structures across tasks. In addition, a close investigation of how improvements in naming relate to improvements in discourse at the individual level would help in better understanding the causes for generalization (ongoing work is focusing on the relationship between naming and discourse improvement behaviorally in the same dataset; den Ouden et al., 2025). Moreover, it should be noted that the general goal of this clinical trial was to uncover predictors for improvement, so there was no group of participants that did not receive treatment, against which the effect of repeated testing could be assessed. Finally, because of the experimental nature of this study, due to the limited evidence on treatment generalization to discourse, this investigation relied on a large number of analyses, which makes it possible that some of the associations between brain and behavior will not survive after replication. Future studies will be able to determine the robustness of the results in new datasets.

### Conclusions

In a large sample of participants with aphasia in the chronic phase, we found that lesions in the dorsal stream are associated with impaired discourse production. Baseline lesions and FA in the temporal lobe are also predictive of generalized discourse improvement after phonological and semantic treatment targeting word production. In particular, we found that the integrity of the hippocampal memory system in the medial temporal lobe is associated with positive performance changes to discourse variables following both types of treatment. Long-term improvements seem to depend on consolidation in the cortex through connectivity via the fornix. The results indicate that the integrity of areas associated with language processing is important for discourse performance at baseline, but for word-level treatment to generalize to spontaneous language production, the ability to learn and integrate incoming information in the existing language network via the hippocampal formation needs to be spared.

## ACKNOWLEDGMENTS

We would like to thank Sara Sayers for her assistance with data collection and scoring.

## FUNDING INFORMATION

Julius Fridriksson, National Institute on Deafness and Other Communication Disorders (https://dx.doi.org/10.13039/100000055), Award ID: P50DC014664.

## AUTHOR CONTRIBUTIONS

**Laura Giglio**: Conceptualization; Data curation; Formal analysis; Visualization; Writing – original draft; Writing – review & editing. **Leonardo Bonilha**: Data curation; Funding acquisition; Writing – review & editing. **Julius Fridriksson**: Funding acquisition; Project administration; Resources; Writing – review & editing. **Sigfus Kristinsson**: Formal analysis; Writing – review & editing. **Roger Newman-Norlund**: Data curation; Methodology; Software; Writing – review & editing. **Chris Rorden**: Data curation; Funding acquisition; Investigation; Methodology; Software; Writing – review & editing. **Brielle C. Stark**: Conceptualization; Writing – review & editing. **Janina Wilmskoetter**: Conceptualization; Writing – review & editing. **Dirk B. den Ouden**: Conceptualization; Data curation; Funding acquisition; Investigation; Project administration; Writing – review & editing.

## CODE AND DATA AVAILABILITY STATEMENT

The neural data is available on OpenNeuro: https://openneuro.org/datasets/ds004884/versions/1.0.1. The discourse data with relative code is on OSF: DOI 10.17605/OSF.IO/SCG6F. All code for neural analyses used is publicly available (https://github.com/neurolabusc/NiiStat).

## Supplementary Material



## TECHNICAL TERMS

Discourse: Usually refers to the output of a task meant to elicit connected spontaneous speech of multiple sentences.
Words per minute (WpM): Measure extracted from discourse task to quantify speech fluency.
Mean length of utterance (MLU): Produced in a discourse task, taken to index sentence processing abilities.
Verbs per utterance (VpU): In a discourse task, used as a proxy for syntactic abilities.
Propositional or idea density (PD): In speech, approximating the amount of information conveyed per number of words.
Tokens: Total number of words produced in a discourse task, used as a measure of gross output.
