# Supplementary material for: Neural Predictors for the Generalization of Semantic and Phonological Treatment to Discourse Performance in Chronic Post-Stroke Aphasia
Source: Neurobiol Lang (Camb). 2025 Dec 18;6:NOL.a.27. doi: 10.1162/NOL.a.27 (PMC12727049; doi:10.1162/NOL.a.27)
Supplement: Supplementary file 1 [file nol-6-1-27-s001.pdf]

Cinderella instructions

From AphasiaBank protocols (https://aphasia.talkbank.org/protocol/english/materials-aphasia/):

Present picture book.

“I’m going to ask you to tell a story. Have you ever heard the story of Cinderella?”

(Make note of answer for demographic data. If answer is no, ask participant to tell a fairy tale s/he knows.)

“Do you remember much about it? These pictures might remind you of how it goes. Take a look at the pictures, and then I’ll put the book away and ask you to tell me the story in your own words.”

Allow participant to look through book (assist with page turning if needed), and then prompt: “Now tell me as much of the story of Cinderella as you can. You can use any details you know about the story, as well as the pictures you just looked at.”

If participant gives a response of fewer than three utterances, or seems to falter, allow 10 seconds, then prompt: “What happened next?” or “Go on.”

Continue until participant concludes story or has clearly finished.

If no response, prompt: “Did Cinderella go to the ball and meet the prince?”

Best Practice Guidelines for Reporting Spoken Discourse in Aphasia and Neurogenic Communication Disorders

The following discourse reporting standards were developed through an expert consensus process conducted as part of a FOQUS Aphasia (www.foqusaphasia.com) initiative. These standards reflect expert opinion at the time they were developed. The authors intend for this to be a dynamic set of recommendations that will shift as the needs and practices within clinical and research communities change. For details regarding the development of these recommendations, or when using these recommendations, cite**:**
Stark, BC & Bryant, L; Themistocleous, H; den Ouden, D-B; Roberts, A (2021). Best Practice Guidelines for Reporting Spoken Discourse in Aphasia and Neurogenic Communication Disorders. *Doi: 10.1080/02687038.2022.2039372.* Visit **https://osf.io/y48n9/** for updates on the project.

**Category**	Item Number	Reporting Standard	Included (Mark 'x')	Page Number(s)
Information about the discourse sample	1	Define "discourse"	x	3
	2	Define "utterance" (or other unit, e.g., turn unit)	x	9
	3*	Number of words in sample	x	10
Information about how the discourse sample was collected	4	Describe elicitation task	x	9
	5	Exact instructions used to elicit discourse sample	x	SI
Information about the persons included in the collection of the discourse sample	6	Demographic information about primary speaker [the person whose discourse is of interest]	x	7-8
	7	Information about the primary speaker's neurological condition	x	7-8
Methodology and rater agreement	8	Inter-rater reliability for each analyzed variable/measure	x	10
	9	Reliability statistics used	x	10
	10	Details on the number (percentage) of files used for determining reliability/agreement	x	10
	11*	Reliability (point to point agreement) for transcription (orthographic or other)		
Analysis	12	Type of transcription (e.g., orthographic, phonetic)	x	9
	13	Detailed description of any perceptual rating scale used, including providing a copy of the scale if not previously published	n/a	
	14	Details of the annotation system, formal (e.g., CHAT) or informal (created by the clinician/examiner)	x	9
	15	Whether transcription was verbatim (e.g., including all behaviors such as fillers) or whether information was excluded in the transcription process.	x	9
	16	Completeness of transcription (full, partial, transcribing errors only)	x	9
	17*	Details of any software used for transcribing/annotating/generating data (e.g., SALT, CLAN, ELAN)	x	9

	18*	Who/what transcribed the sample (by a human, by a machine/software, hybrid human and software)	x	9
Information about the individual discourse variables/behaviors reported	19	What is being used as primary outcome measure(s) (e.g., linguistic information, speech information, etc)	x	3,9
	20*	Theoretical rationale for selecting variable/behavior/outcome measure(s)	x	3
	21	Operational definition for each variable/behavior/outcome(s)	x	3
